# Advancement of the Power-Law Model and Its Percolation Exponent for the Electrical Conductivity of a Graphene-Containing System as a Component in the Biosensing of Breast Cancer

**DOI:** 10.3390/polym14153057

**Published:** 2022-07-28

**Authors:** Yasser Zare, Kyong Yop Rhee, Soo Jin Park

**Affiliations:** 1 Biomaterials and Tissue Engineering Research Group, Department of Interdisciplinary Technologies, Breast Cancer Research Center, Motamed Cancer Institute, ACECR, Tehran 1125342432, Iran; 2 Department of Mechanical Engineering (BK21 Four), College of Engineering, Kyung Hee University, Yongin 17104, Korea; 3 Department of Chemistry, Inha University, Incheon 22212, Korea

**Keywords:** graphene, polymer nanocomposite, percolation theory, conductivity, interphase, tunneling distance

## Abstract

The power-law model for composite conductivity is expanded for graphene-based samples using the effects of interphase, tunnels and net on the effective filler fraction, percolation start and “b” exponent. In fact, filler dimensions, interphase thickness, tunneling distance and net dimension/density express the effective filler fraction, percolation start and “b” exponent. The developed equations are assessed by experimented values from previous works. Additionally, the effects of all parameters on “b” exponent and conductivity are analyzed. The experimented quantities of percolation start and conductivity confirm the predictability of the expressed equations. Thick interphase, large tunneling distance, high aspect ratio and big nets as well as skinny and large graphene nano-sheets produce a low “b” and a high conductivity, because they improve the conduction efficiency of graphene nets in the system. Graphene-filled nanocomposites can be applied in the biosensing of breast cancer cells and thus the developed model can help optimize the performance of biosensors.

## 1. Introduction

Carbon nanotubes (CNT) have a tubular structure of carbon atoms [1,2,3,4,5,6,7,8,9]. However, graphene 2D nano-sheets in the form of sp^2^ carbon show wonderful electronic, unique mechanical, significant thermal and good chemical properties [10,11,12,13,14,15,16,17,18]. Thus, polymer nanocomposites containing graphene can be applied in different technologies such as transparent electronics, electromagnetic interference shielding, energy devices, light emitting diodes and lightning protection [19,20,21,22,23]. These applications mainly need to the electrical conductivity justifying the wide research on the conductivity graphene-filled products. The conductivity in nanocomposites is achieved when the filler percentage reaches an essential level as percolation start [24,25,26]. Actually, the significant effect of graphene on the conductivity is obtained after percolation start and the formation of conductive graphene nets. The polymer nanocomposites containing graphene nano-sheets present lower percolation start and more conductivity compared to CNT systems [27], because the graphene has big aspect ratio and very giant specific superficial zone. However, some undesirable phenomena such as aggregation, crimping and difficult networking may weaken the efficiency of graphene for conductivity [28].

The conductivity of graphene-based polymer systems has been extensively studied by experimental studies [29,30,31]. They focused on the physical and processing factors to obtain the little percolation start and promote the conductivity by low volume fraction of graphene. However, the influence of main terms on the percolation start and conductivity of graphene-filled samples was not studied. This matter can be evaluated by the modeling methods, but the theoretical studies of graphene nanocomposites mainly include the application of the simple power-law model to calculate the percolation start and conductivity [29,32,33]. So, the roles of main parameters such as nano-dimensions and interphase regions in the conductivity graphene systems were not evaluated.

The interphase regions are commonly formed in nanocomposites, due to the large interfacial zone amongst polymer medium and nanofiller [34,35,36,37,38,39]. The characters of interphase dimensions and toughness in the rigidity of nanocomposites have been conferred in previous articles [40,41,42,43,44,45,46]. Furthermore, it was indicated that the interphase regions can facilitate the production of conductive nets in the samples in advance the real attachment of particles [47,48,49,50,51]. Thus, the interphase regions can positively decrease the percolation level to low filler fractions. However, the conventional model such as the power-law model cannot consider the important issues in nanocomposites such as nanoscale, interphase and tunnels.

The power-law model shows respectable arrangement with the experimented conductivity of graphene products [32,52]. Nonetheless, this model disrespects the main attributes of graphene nanocomposites. Additionally, there is no accurate equation for the “b” exponent in this model. In this work, this conventional model is advanced for graphene-filled systems assuming the impacts of interphase, tunnels and the dimensions/density of filler nets on the effective filler fraction, percolation start and “b” exponent. The established equations are assessed using experimental results from previous papers. Likewise, the impact of all factors on the “b” exponent and conductivity is analyzed to confirm the advanced technique.

## 2. Theoretical Views

The simple power equation for calculating the conductivity of composites was suggested [29] as:

(1) $$\sigma =\sigma_{f}{\left(\phi_{f}-\phi_{p}\right)}^{b}$$

where “σ_f_” is the filler conduction, “$\phi_{f}$” is the filler volume portion, “$\phi_{p}$” is the volume share at percolation start and “b” is the exponent. Additionally, “b” was reported to be 1.6-2 and 1-1.3 for 3D and 2D systems, respectively [52], although more “b” value was calculated for polymer graphene nanocomposites.

This equation only reflects the effects of conduction, amount and percolation start of particles on the conductivity, but it neglects the interphase and tunneling zones, as mentioned. Undoubtedly, these terms affect the effective volume fraction and percolation start of nanofiller, which change the conductivity of whole system.

The interphase zones of the nanoparticles increase the efficiency of nanofiller, because they can decrease the distance between nano-sheets and contribute to the net. The interphase volume portion in graphene-based nanocomposites [53] is predicted by:

(2) $$\phi_{i}=\phi_{f}(\frac{2t_{i}}{t})$$

where “t” and “t_i_” are the thicknesses of the nano-sheets and interphase, respectively.

The effective graphene volume portion in the samples can be calculated by the total sum of the interphase and filler as:

(3) $$\phi_{eff}=\phi_{f}+\phi_{i}=\phi_{f}(1+\frac{2t_{i}}{t})$$

which highlights that the interphase thickness and graphene thickness control the effectiveness of nanoparticles in the nanocomposite.

The percolation start of 3D unsystematically organized graphite sheets in the nanocomposite was also recommended [31] as:

(4) $$\phi_{p}=\frac{27\pi D^{2}t}{4{\left(D+d\right)}^{3}}$$

“D” is the diameter of the nano-sheets and “d” is the tunneling distance. Nonetheless, D >> d eliminates the role of tunnels in the latter equation as:

(5) $$\phi_{p}=\frac{27\pi t}{4D}$$

The interphase regions are formed on both sides of the graphene nano-sheets. In addition, the tunneling spaces comprise the distance between the adjacent nano-sheets. The impacts of interphase and tunnels on the percolation start can be suggested by the development of the above equation as:

(6) $$\phi_{pi}=\frac{27\pi t}{4D+2(Dt_{i}+Dd)}$$

The predictability of this equation for the percolation start of polymer graphene nanocomposites is examined by the tentative facts in the next section.

Assuming the graphene aspect ratio (α = D/t), “$\phi_{pi}$” is given by:

(7) $$\phi_{pi}=\frac{13.5\pi }{\alpha (2+t_{i}+d)}$$

expressing an opposite relation amid percolation start and aspect ratio.

The “b” exponent was insufficiently defined in the previous articles for polymer composites and nanocomposites. Some authors have correlated the “b” to particle diameter and distribution [54,55]. Shao et al. [56] also defined the “b” as a function of universal critical exponent, a structure factor and the number fractions of hanging ends and backbone framework. More recently, Mutlay and Tudoran [30] have developed the Shao approach and suggested that the “b” exponent depends on the dimensional particle distribution, structure factor and aspect ratio of nanoparticles. They yielded good agreement between the predictions and experimental data in graphene and graphite nanocomposites [30]. However, their equation does not assume the interphase and tunnels as well as net dimensions, which undoubtedly affect the “b” exponent.

The “b” exponent can be defined for graphene samples by the mentioned terms by mathematical operations as:

(8) $$b=4+\frac{10}{t_{i}+1}+\frac{10}{d+1}+\frac{500}{\alpha }-\frac{N}{5}$$

where “N” shows the dimensionality, dimension and density of filler nets in the nanocomposite. The correctness of this equation is also examined in the next section by the tested data of conductivity in dissimilar examples.

Supposing the impacts of interphase and tunnels on the effective graphene amount (Equation (3)), percolation start (Equation (7)) and “b” exponent (Equation (8)), the power-law model in Equation (1) is:

(9) $$\sigma =\sigma_{f}{\left(\phi_{eff}-\phi_{pi}\right)}^{4+\frac{10}{t_{i}+1}+\frac{10}{d+1}+\frac{500}{\alpha }-\frac{N}{5}}$$

**Figure 1:**
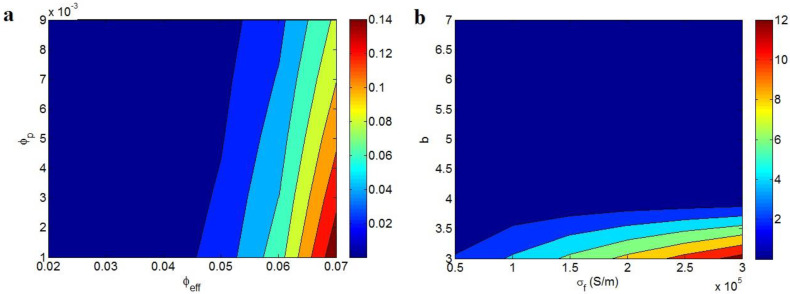
Two-dimensional plots showing the estimates of conductivity using Equation (9) at (**a**) various ranges of “$\phi_{eff}$”and “$\phi_{p}$” (σ_f_ = 10^5^ S/m and b = 5) and (**b**) different levels of “σ_f_” and “b” ($\phi_{eff}$ = 0.04 and $\phi_{p}$ = 0.005).

Figure 1 depicts the effects of various factors on the forecasts of this model. In Figure 1a, the best conductivity is obtained as 0.14 S/m at $\phi_{eff}$ = 0.07 and $\phi_{pi}$ = 0.001, while low “$\phi_{eff}$” significantly decreases the conductivity. Figure 1b also reveals that the greatest conductivity of 12 S/m is found by σ_f_ = 3 × 10^5^ S/m and b = 3, whereas the conductivity mainly falls at b > 4. As a result, the highest nanocomposite conductivity is gained by the uppermost grades of effective filler fraction and graphene conduction as well as by the smallest ranges of percolation start and “b” exponent. Moreover, it is observed that both filler conduction and “b” affect the conductivity more compared to other parameters.

The developed model assumes the influence of graphene agglomeration on the conductivity when the average dimensions of agglomerations are considered. The agglomerates of graphene have different sizes and aspect ratios from a graphene layer, which affect the effective graphene concentration (Equation (3)), percolation start (Equation (7)), “b” (Equation (8)) and conductivity (Equation (9)). Therefore, it is possible to take into account the agglomeration of graphene in the conductivity of nanocomposites using the developed model.

## 3. Results and Discussion

### 3.1. Assessment of Equations by Experimented Records

The obtained equations for percolation start, “b” and electrical conductivity are assessed using the experimental facts of graphene systems from literature.

Table 1 shows the reported specimens and the levels of “t”, “D” as well as “$\phi_{p}$” from the measurements of electrical conductivity at different filler concentrations. By comparing the experimental “$\phi_{p}$” to Equation (6), the values of “t_i_” and “d” are calculated and observed in Table 1. The dissimilar values of “t_i_” and “d” show the existence of unlike interphase and tunnels in the examples. The densest interphase (8 nm) and the largest tunnels (10 nm) are witnessed in samples No. 4 and 3, respectively. It should also be indicated that disregarding these parameters results in the incorrect estimation of percolation start. In other words, only the geometries of graphene nano-sheets cannot yield the very small percolation start in nanocomposites, but the interphase around the nanoparticles and the tunneling spaces between neighboring nano-sheets play a role in the percolating of nanoparticles. Accordingly, Equation (6) finely predicts the percolation start in graphene-filled nanocomposites, considering the impacts of the interphase and tunneling zones.

**Figure 2:**
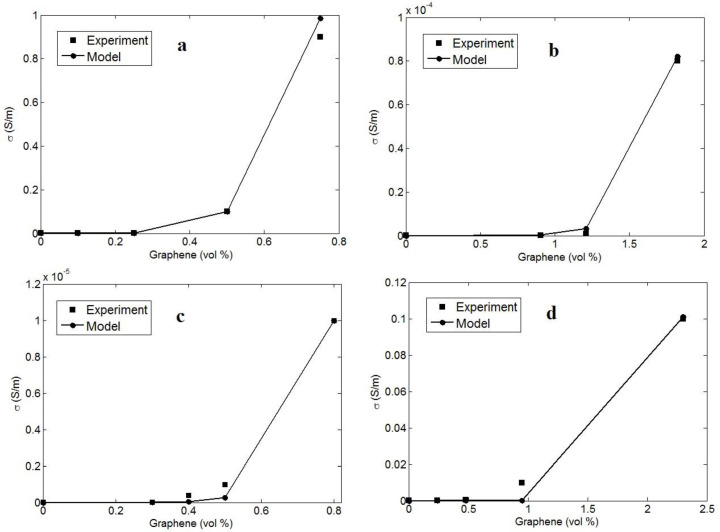
The experimented and calculated conductivity using the novel model for (**a**) PS [59], (**b**) epoxy [60], (**c**) PVDF [29] and (**d**) ABS [61] graphene systems.

The tested conductivity of the examples is applied to the innovative model and the values of the “b” exponent are calculated. Figure 2 shows the tested conductivity and the model’s calculations for the examples. The model’s estimates acceptably agree with the tested results. Thus, it is logical to apply the developed form of the power-law model (Equation (9)), supposing the interphase and tunnels for the approximation of conductivity in the graphene systems. The calculated values of “b” for the reported samples are shown in Table 1. The smallest and the highest levels of “b” are obtained as 4 and 7.5 for samples No. 1 and 9, respectively. As a result, “b” changes from 4 to 7.5 for the examples. This range is greater than the values of “b” calculated for graphene nanocomposites using the conventional power-law model (Equation (1)), disregarding the interphase and tunneling parts.

The values of the “b” exponent can be applied using Equation (8) to approximate the level of “N”. The calculated “N” for all samples is reported in Table 1. “N” ranges from 1.5 to 22 for the reported examples. As recommended, “N” is a representative of the dimension and density of filler net. So, a higher “N” shows the creation of bigger and thicker nets in the sample. It can be suggested that sample No. 2 contains the biggest and the densest nets among the reported samples. Additionally, an inverse relation between “N” and “b” is extracted from the reported calculations in Table 1. The samples with high “N” illustrate small “b”, while a low “N” results in a high “b”. This evidence is logical, because the big and dense nets of graphene produce a strong conductivity in the nanocomposite as predicted by low “b” (see Figure 1b). Conclusively, Equation (8) successfully states the possessions of interphase depth, tunneling distance and net dimensions on the “b” exponent. In other words, the suggested equation for “b” considers the influence of all main factors, which may govern the percolation start and the nets of graphene nano-sheets in the nanocomposite. In the absence of accurate experimental techniques for the characterization of interphase, tunneling and net dimensions/density, the developed equations for percolation start and “b” exponent in this study can help approximate these parameters in polymer graphene nanocomposites.

### 3.2. Parameters’ Effects on the “b”

The stimuli of parameters on the “b” exponent are discussed using Equation (8).

**Figure 3:**
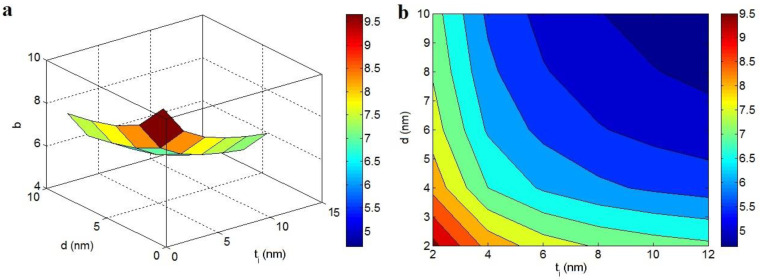
Effects of “t_i_” and “d” on “b” at normal t = 2 nm, D = 1 μm and N = 10 by (**a**) 3D and (**b**) 2D pictures.

Figure 3 exemplifies the characters of “t_i_” and “d” in the “b” at t = 2 nm, D = 1 μm and N = 10. The highest value of “b” as 9.5 is observed at t_i_ = d = 2 nm, while “b” decreases to about 4.15 at t_i_ > 10 nm and d > 8 nm. Consequently, the high values of both “t_i_” and “d” decrease “b”. In other words, thick interphase and long tunneling distance can produce a low “b”, whereas thin interphase and short tunneling distance undesirably enhance it.

It was mentioned that the “b” exponent inversely depends on the properties of graphene nets in the nanocomposite. Both “t_i_” and “d” significantly affect the mentioned terms. The interphase adjoining the nanoparticles can participate in the filler nets; thus, they facilitate the percolation of nanoparticles and enhance the size and compactness of the nets. Likewise, the tunneling spaces between adjacent nanoparticles can contribute to the networking of graphene nano-sheets, because the nanoparticles can form the nets in the presence of tunneling regions [62,63]. As a result, thick interphase and large tunneling distance can raise the scale and density of conductive nets in the nanocomposites, which diminishes the “b”.

**Figure 4:**
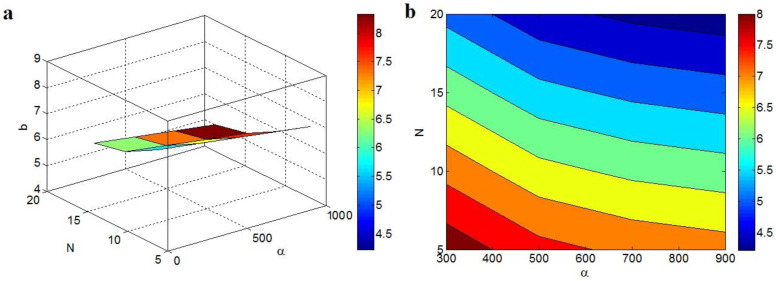
Variations in the “b” exponent at dissimilar grades of “α” and “N” and t_i_ = 4 nm and d = 5 nm by (**a**) 3D and (**b**) 2D schemes.

“b” exponent at different values of “α” and “N” and average t_i_ = 4 nm and d = 5 nm are also depicted in Figure 4. The high levels of both “α” and “N” decrease the “b”, but the highest “b” is projected by the minimum values of these factors. As shown, α = 900 and N = 20 produce b = 4.2, while b = 8 is obtained by α = 300 and N = 5. So, the high levels of these parameters can positively reduce the “b”, highlighting that the large aspect ratio and high net properties can produce a desirable “b”.

The associations of “b” to these factors are expected, due to the direct influence of “α” and “N” on the performance of graphene nets in the nanocomposite. As predicted, a high aspect ratio of the nano-sheets can produce a slight percolation start, which desirably affects the magnitudes of the nets [60,64]. In fact, the large aspect ratio of the nanoparticles improves the scale and density of the conductive nets. Conversely, a high rank of “N” obviously increases the net properties, because “N” reveals the dimensions/density of nets. Thus, a small “b” is observed due to the big aspect ratio and “N”.

**Figure 5:**
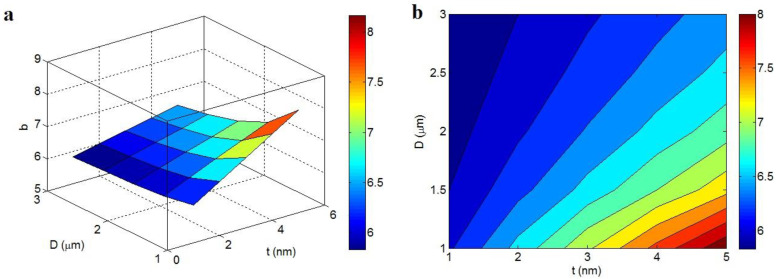
Dependencies of “b” exponent on “t” and “D” at t_i_ = 4 nm, d = 5 nm and N = 10 by (**a**) 3D and (**b**) 2D charts.

The calculations of the “b” exponent at unlike arrays of “t” and “D” are also seen in Figure 5. A small “t” and large “l” decrease the “b” exponent. As shown, t = 5 nm and D = 1 μm result in b = 8, while b = 5.8 is achieved by t < 1.5 nm and D > 2.5 μm. It can be suggested that the thin and large graphene nano-sheets positively influence the “b”, while thick and small nano-sheets detrimentally affect it. So, it is necessary to control the dimensions of graphene nano-sheets in the nanocomposite to obtain a good “b”.

Skinny and large nano-sheets beneficially manage the size of nets in the products, because they cause a poor percolation start and also produce dense nets. At a constant volume of nanoparticles, thin nano-sheets show a high number. Therefore, the nanocomposites containing thinner nano-sheets contain a larger number of nanoparticles. Additionally, the contacts between larger nano-sheets are more than those of short ones. Thus, skinny and big nano-sheets can develop the attributes of filler nets in the nanocomposite; therefore, the developed equation accurately forecasts the “b”.

### 3.3. Parameters’ Effects on the Conductivity

The impact of the parameters on the conductivity of graphene is evaluated by the developed equation (Equation (9)) based on interphase and tunneling regions. In all calculations, σ_f_ = 10^5^ S/m is considered.

**Figure 6:**
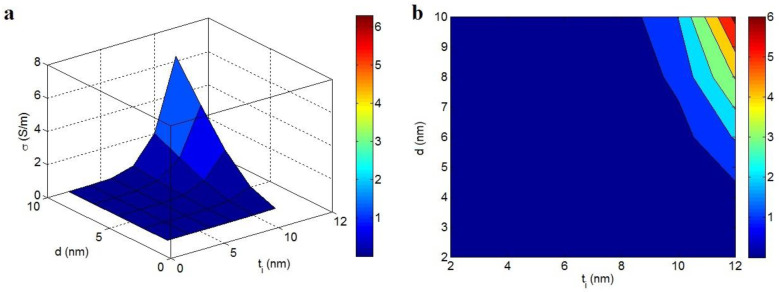
Impact of “t_i_” and “d” on the nanocomposite’s conductivity using (**a**) 3D and (**b**) 2D designs.

Figure 6 shows the conductivity of the nanocomposite correlating to “t_i_” and “d” at t = 2 nm, $\phi_{f}$ = 0.01, D = 1 μm and N = 10. The top conductivity as 6 S/m is witnessed at the extreme levels of t_i_ = 12 nm and d = 10 nm. However, t_i_ < 8.5 nm and d < 5 nm induce very little conductivity adjacent to 0. So, profuse interphase and high tunneling distance can harvest a high conductivity. Instead, thin interphase and short tunnels cannot significantly improve the conductivity.

As mentioned, the interphase and tunnels take part in the nets because they surround the nanoparticles. Therefore, profuse interphase and large tunnels significantly increase the net size/density, which positively improves the conductivity. It can be said that a thick interphase and a large tunneling space can involve more nanoparticles in the conductive nets. In contrast, thin interphase and short tunneling distance negligibly manipulate the net size/density. So, the advanced model shows reasonable impacts of interphase deepness and tunnel size on the nanocomposite’s conductivity. However, it should be said that a very large tunneling distance between adjacent nano-sheets weakens the tunneling effect, producing insulation.

**Figure 7:**
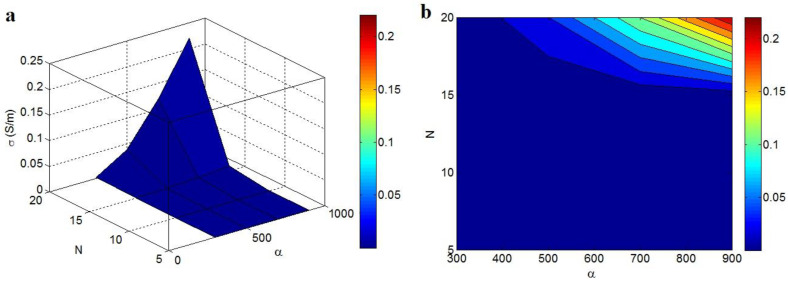
Impact of “α” and “N” on the conductivity by (**a**) 3D and (**b**) 2D schemes.

The impacts of “α” and “N” on the conductivity of the system at t = 2 nm, t_i_ = 4 nm, $\phi_{f}$ = 0.01, and d = 5 nm are also illustrated in Figure 7. The finest results are gained by the peak values of “α” and “N”, though a pitiable conductivity is witnessed at low levels of these factors. The upper conductivity of 0.22 S/m is calculated at α = 900 and N = 20, while N < 15 only decreases the conductivity to about 0. As a result, only a low level of “N” can decrease the conductivity, but the highest ranges of both the aspect ratio and “N” produce the highest conductivity.

The optimistic role of the aspect ratio in the sample’s conductivity is attributed to its impacts on the percolation start and net dimensions. A high aspect ratio results in a low percolation start in the graphene nanocomposites, as mentioned. Additionally, a high aspect ratio causes a big surface zone, which creates large nets. A low percolation start boosts the conductivity of the system, as seen in Figure 1a. Moreover, the efficiency of electron transferring throughout the nanocomposite is improved by the formation of large nets. Consequently, the correlation of conductivity to the aspect ratio is logical. In addition, the “N” shows the magnitude of filler nets in the nanocomposite. A small “N” shows the foundation of short and weak nets in the specimens, whereas a high “N” depicts the large and dense nets. Therefore, “N” rightly manages the conductivity of the graphene nanocomposite, as recommended by the new methodology.

**Figure 8:**
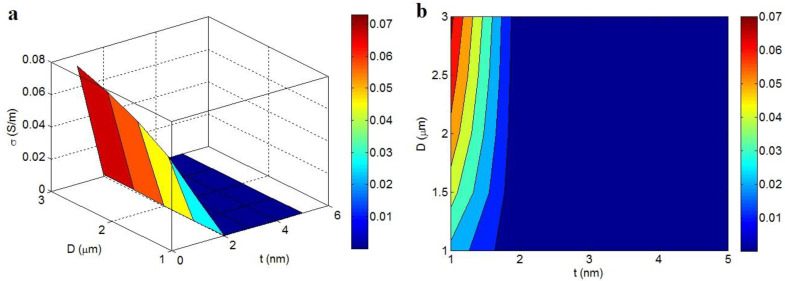
(**a**) Three-dimensional and (**b**) two-dimensional charts for the characters of “t” and “D” in the conductivity.

Figure 8 also reveals the influence of graphene dimensions on the conductivity of nanocomposites (t_i_ = 4 nm, $\phi_{f}$ = 0.01, d = 5 nm and N = 10). t > 2 nm reduces the conductivity to about 0, but the smallest “t” (t = 1 nm) and the highest “D” (D = 3 μm) harvest the uppermost conductivity as 0.07 S/m. Accordingly, the best conductivity is obtained by very thin and large graphene nano-sheets. On the other hand, thick nano-sheets cannot increase the conductivity of the nanocomposite.

The thin and large nano-sheets control the percolation start and net scale in the nanocomposite based on Equations (6) and (8). Additionally, polymers are frequently insulated and the conductive nanofillers handle the whole conductivity. Since the conductivity of the nanocomposite depends on the percolation level and the net dimensions, skinny and large nano-sheets can play an optimistic role in the conductivity. In fact, the thin and large nano-sheets can cover a high fraction of the nanocomposite, which produces big nets and high conductivity. Alternatively, the efficiency of dense and small nano-sheets is insignificant, because they produce small nets, which cannot effectively transfer the electrons. As a result, the optimistic effects of skinny and large nano-sheets on the conductivity of samples are meaningful.

## 4. Conclusions

The power equation for composite conductivity was developed for graphene-filled samples, determining the effects of interphase, tunnels and net dimension/density on the effective filler fraction, percolation start and “b” exponent. Additionally, the measured records of percolation start and conductivity were applied to confirm the predictability of the established equations. A high conductivity is found using a large filler amount, slight percolation start, significant filler conduction and small “b”; however, the impact of the filler conduction and the “b” exponent is more significant compared to the other parameters. The experimental data of percolation start have good arrangement with the predictions. So, the interphase depth and tunneling size play a main role in the percolation value of graphene in the system. Moreover, the innovative model adequately predicts the conductivity of the examples. Generally, thick interphase, large tunneling distance, high aspect ratio and dense nets as well as thin and big graphene nano-sheets produce a low “b” exponent. In addition, these factors cause high conductivity, suggesting that they considerably increase electron transfer in the system. This model was only developed for polymer graphene nanocomposites. Since graphene-filled nanocomposites can be used in the biosensing of breast cancer cells, the developed model can help enhance the performance of biosensors.

## Figures and Tables

**Table 1:** Selected examples and the outputs of numerous terms by the equations.

No.	Samples [Ref.]	t (nm)	D (μm)	*ϕ_p_*	t_i_ (nm)	d (nm)	N	b
1	PI ^1^/graphene [57]	3	5	0.0015	7	9	13.0	4.0
2	PET ^2^/graphene [58]	2	2	0.0050	3	4	22.0	4.6
3	PS ^3^/graphene [33]	1	4	0.0005	7	10	7.00	4.9
4	PS/graphene [59]	1	2	0.0010	8	8	4.50	5.6
5	PVA ^4^/graphene [52]	2	2	0.0035	5	5	10.5	5.7
6	epoxy/graphene [60]	2	2	0.0050	2	4	14.5	7.0
7	PVDF ^5^/graphene [29]	1	2	0.0030	2	3	15.5	7.0
8	SAN ^6^/graphene [61]	1	2	0.0017	5	5	1.50	7.3
9	ABS ^7^/graphene [61]	1	4	0.0013	3	3	8.00	7.5

^1^: polyimide; ^2^: poly (ethylene terephthalate); ^3^: polystyrene; ^4^: poly (vinyl alcohol); ^5^: poly (vinylidene fluoride); ^6^: acrylonitrile butadiene styrene; ^7^: styrene acrylonitrile.

## Data Availability

All data are available in the paper.
